# Clinical Effects of Yoga on Asthmatic Patients: A Preliminary Clinical Trial

**Published:** 2010-07

**Authors:** Demeke Mekonnen, Andualem Mossie

**Affiliations:** 1 Department of Pathology, Jimma University; 2 Department of Physiology, Jimma University

**Keywords:** Asthma, Yoga, Expiratory flow rate, Salbutamol, Ethiopia

## Abstract

**Background:**

Asthma is one of the commonest respiratory diseases in Jimma area as well as a significant disease burden worldwide costing billions of dollars. Anti-asthmatic drugs that are available in the market are expensive and have adverse effects. Thus, it is wise to look for an adjunct therapy to alleviate these problems. Therefore, the main aim of this study is to see the effect of yoga on patterns of clinical features, peak expiratory flow rates and use of drugs in asthmatic patients.

**Methods:**

A preliminary controlled clinical trial study was conducted on 24 volunteer asthmatic patients who were getting support at the missionary of charity. They were grouped in yoga and control groups. An Indian yoga expert through a translator conducted the training on yogic practice, yogic posture, breath slowing technique and discussion at the end. Then, the yoga groups were supervised for four weeks taking yoga exercise daily for 50 minutes. Peak expiratory flow rate was taken using the mini Wright peak flow meter and vital signs were measured in both groups. Data were analyzed using web based Graph pad quick calcs statistical software.

**Results:**

The male to female ratio was 1:1 in both cases and control groups, 8(66.7%) were Christian and 9 (75.0%) were farmers. The yoga group showed 66.7% reduction in the use of salbutamole puff and 58.3% salbutamole tablets. There was a 10% increment in the PEFR in the yoga group while only 2% in the control group. There was statistically significant reduction in day and night asthma attacks in the yoga group.

**Conclusion:**

Yoga exercise among asthmatic patients resulted in a decreased number of day and night attacks and use of drugs. It also shows significant improvement in the peak expiratory flow rate. Further large scale study is recommended.

## Introduction

Asthma is a chronic (long-term) lung disease that inflames and narrows the airways by spastic contraction of the smooth muscle in the bronchioles, which partially obstructs the bronchioles and causes recurring periods of wheezing, chest tightness, shortness of breath, and coughing (1,2). Though the prevalence and incidence of asthma is difficult to assess with certainty because of lack of reliable population based figures which used uniform diagnostic criteria, however, it has been suggested that approximately 5% of adult and 7–10% of children in USA and Australia have the disorder (4). According to the CDC (center for disease control) report, 10–11 million persons had acute attacks in 1998, which results in 13.9 million out patient visits, 2 million request for urgent care, and 423,000 hospitalizations, with a total cost of >6 Billion USD (3,4). In Africa, it accounts for 2–10% of medical admissions (5). In South West Ethiopia asthma accounted for 2% of out patient and 5.4% of medical admission (6–8).

Yoga, or its ancestor, first appeared some where around 5,000 years ago. Since then, it has gradually evolved in to the modern form. The word “Yoga” means union, joining or to link together as one whole according to Sansacrit language. Hath yoga is a physical method which uses the breath to link the various parts of the body and the mind and to allow them to behave as one functional unit which helps in the control of Asthma (9–11).

Different studies have revealed that yoga has significant importance in improving symptoms of asthmatics and in hypertension (12–18). It was found that one preliminary study of yoga therapy for asthma on 46 patients data clearly shows a significant qualitative improvement in the severity and duration of attacks; attacks per week and decrease medication use (19).

Significant symptomatic improvement after yoga training for 9 months in patients with chronic severe airways obstruction was observed in Australia. A decade ago both short and long-term prospective studies in India showed clearly the beneficial effects of yoga in the management of bronchial asthma (20). A study showed significant effect with in short time (13 days) of yoga practice (21).

Since bronchial asthma is an important cause of morbidity and mortality especially in resource limited areas where the long term use of multiple drugs is costly, it is wise to think alternative way to treat such an illness with better economic safety and avoid adverse effect of the drugs. The study therefore tries to look into the applicability of yoga as an alternative approach in the treatment of asthma in Ethiopia. The research so conducted aimed at determining the clinical features, peak expiratory flow rate and use of drugs for their asthma in asthmatic patients before and after the yoga practice.

## Patients and Methods

The study was conducted at the missionary of charity in Jimma town, southwest Ethiopia from March 28 to April 27, 2009 GC. A clinical trial, controlled prospective study was conducted by randomly dividing patients into experimental/intervention (yoga group) and control group. Yoga exercise was given every day for 50 minutes for four weeks by a yoga trainer.

The training included;

1. Integrated yogic practice: initial warm up activity with relaxed breathing technique with stretching exercise breathing exercise (5minute).
  - Loosening exercise (5 minute) yoga practice to loosen various joints.
2. Yogic Postures
  - General physical postures (10 minute) simple easy physical postures in standing and sitting along with specific slow breathing.
  - Deep relaxation practice (10 minute) to consciously relax muscles followed by conscious slowing of breathing and calming of the mind.
3. Breath slowing technique (10minute) performed with easy comfortable and slow deep breathing with out voluntary breath holding.
4. Discussion (10 minute) to understand the emotional wellbeing and positive out look in general and to result in a feeling of freedom and relaxation.

Twenty four patients diagnosed to have asthma and being supported in the Missionary of Charity who volunteered to take part in the study participated.

***Inclusion criteria*:** Patients who are diagnosed at hospital level to have bronchial asthma; patients who are on regular follow up at chest clinic; patient with mild to moderate asthma and those who were able to come to missionary of charity for the yoga practice were included in the study based on their consent.

***Exclusion criteria*:** patients who were not volunteer to abide by the agreement; patients with chronic obstructive lung disease; patients with associated lung disease (diagnosed to be with active tuberculosis; patients with severe asthmatic attack since they can not sit comfortable to attend the yoga practice; patients who are diagnosed to have cardiac disease were excluded.

Background information about their asthma and experiences was collected on structured questionnaire prior to the intervention. Same information was collected during the intervention and after 4 weeks. A physician who was blinded to the groups helped to complete the questionnaire and conducted the peak expiratory flow meter test.

Physical examination and objective peak flow meter evaluation before and 30 minute after the yoga exercise were done. A mini Wright peak flow meter was used; using the standard value percentage was calculated and average was taken to compare the mean changes.

Data were cleared and using a web based soft ware (statistics for health Graph Pad Quick Calcs and chi square calculator) chi square, p-value and paired students t-test were analyzed.

The study was approved by the medical sciences faculty of Jimma University. Participants were given information about the study objective, voluntary participation and told to their treatment. They were also told about the activities that are going to be practiced and were also informed as they can withdraw from participation at any stage. Those who signed the consent form participated in the study.

Literate was defined as able to read and write otherwise illiterate.

## Results

Twenty four patients who fulfill the inclusion criteria and signed the consent randomly categorized as yoga and control group. The male to female ratio was 1:1 in each group, mean age was 30 years for yoga and 31 for control group and most of them were farmers (Table 1).

**Table 1:** Socio-demographic characteristics of asthmatic patients at the Missionary of Charity Jimma Town, Ethiopia 2009.

Socio-demographic features		Frequency	

		Yoga group	Control group
Sex	Male	6	6
	Female	6	6
Age Mean (Range)		30(11–51)	31(12–50)
Religion	Christian	8	7
	Muslim	4	5
Ethnicity	Oromo	7	7
	Amhara	3	4
	Kefficho	2	1
Occupation	Student	3	4
	Farming	9	8
Education level	Illiterate	6	5
	Literate	6	7

The mean age since diagnosis was 4 years in the both groups. The yoga group had follow up for 2 years while the control group for 3 years. All subjects in both groups were diagnosed to have mild persistent asthma and were on treatment (Table 2).

**Table 2:** Previous history of their asthma of asthmatic patients at the Missionary of Charity Jimma Town, 2009.

Past history of Asthma	Frequency	

	Yoga group	Control group
Duration after diagnosis; Mean (range) years	4 (1–10)	4 (1–8)
Duration of follow up; Mean (range) years	2 (1–5)	3 (0–5)
Tablets taken per week (mean)	14	14
Puff use per week(mean)	8	7
Day time attack per week(mean)	4	5
Night time attack per month (mean)	3	3

There was a significant decrement in the number of asthma attacks (day and night) and use of drugs especially to the use of puff (p = 0.044) in the yoga group during and after the 4 week exercise. Eight (66.7%) of the yoga group reduced use of salbutamole puff. In the control group the reduction was in the use of puff was only in 16.6% (Table 3).

**Table 3:** Effect of yoga on the use of salbutamole puff and tablet among asthmatic patients throughout the follow up time at the missionary of charity Jimma 2009.

Change in drug use		Yoga group (n=12)	Control group (n=12)	X^2^	P-value
Salbutamol tablet					
	Reduced	7(58.3%)	1(8.3%)		
	No change	2(16.7%)	3(25%)	7.672	0.021
	Increased	3(25%)	9(66.7%)		
Salbutamol puff					
	Reduced	8(66.7%)	2(16.65%)		
	No change	3(25%)	8(66.7%)	6.206	0.044
	Increased	1(8.3%)	2(16.65%)		

Most of the subjects in the yoga group, showed a decreased number of day attacks per week and night attacks per month as compared to the control group (p = 0.013). Mean change in the PEFR was 10 in the yoga group whereas 2 in the control group which was statistically significant (p <0.0001). Similar pattern was also observed in the mean change of pulse rate, respiratory rate and wheezing among the two groups (P <0.001) (Table 4).

**Table 4:** Mean changes in the clinical features and peak expiratory flow rate before and after the yoga exercise in asthmatic patients at the Missionary of Charity, Jimma 2009.

Mean Changes		Yoga group (n=12)	Control group (n=12)	*P-value
Pulse Rate				
	Before	82	83	<0.0001
	After	74	81	
Respiratory rate				
	Before	24	23	<0.001
	After	20	23	
Wheezing present				
	Before	10	10	<0.001
	After	6	10	
PEEFR				
	Before	72	73	<0.0001
	After	82	75	
Day attacks				
	Before	4	5	<0.001
	After	2	5	
Night attacks				
	Before	2	3	<0.001
	After	2	3	

* Paired student's t-test for matched groups for each mean.

## Discussion

This study showed a reduction in asthma attacks in the yoga group which is comparable to the reports from India (19, 22) and Indonesia (20). This is an evidence for the effect of yoga in helping the co-ordination of breath and movement associated with good posturing for best relaxation of breath muscles. It also helps in controlling the panic attacks which aggravate individual's further deterioration and shortness of breath by letting a way to control physical body, the mind (Psycho somatic) and the autonomic nature of breath control.

The decrease in the number of attacks (day and night) resulted in the reduction of use of asthma drugs especially salbutamol puff which is comparable to the study in India where it showed 69% decrement in the use of oral asthma medications (21, 22) for the acute attacks. The improvement in peak expiratory flow rate in this study is also comparable to the Indian studies (19,22). Change in respiratory rate and pulse rate is also comparable to reports from Indian studies (19, 21). This explains the effect of yoga in the relief of asthma attack and improving the quality of life. The responsiveness of air ways is noticeably increased in asthma patients so that they develop broncho-constrictions for smaller amount of physicochemical stimuli than the healthy ones. There is a complex interplay of several factors: inherent responsiveness of smooth muscles, abnormality in autonomic nervous control and breakdown in airway defenses may promote bronchial hyper reactivity. So, reducing the hyper-responsiveness of the patients will benefit them to have good outcome in the control of their asthma. The other way is the psychological effect on asthma progression, though the mechanism is complex and not well understood; still psychological factors affect about half of all patients. This was the focus pointed to be improved by the yoga exercise and shown improvement at different studies (19–22).

The above effect could therefore be, as different authors (9–11) claimed that psycho-somatic imbalance is present in many, if not all, patients with asthma. Suppressed emotion, anxiety, dependence, and extreme self consciousness may all be accompanied by generalized and localized muscle tension, including that of the voluntary respiratory musculature. This increased muscle tension may be a precipitating or concomitant factor that perpetuates and aggravates the asthmatic syndrome. Yoga seems to stabilize and reduce the excitability of nervous system. Therefore it can reduce efferent vagal reactivity, which has been recognized as the mediator of the psychosomatic factor in asthma.

As life is kept as it is through the process of breathing, any of the ailments in the three processes (inhalation, retention and exhalation) will hamper the quality of life. One of the ailments especially during exhalation is bronchial asthma. This study therefore, comes to action to forward a new approach of cost effective treatment and the following conclusion is drawn.

In conclusion, this study showed that Yoga decreased the number of day and night asthma attacks, use of drugs especially salbutamol puff and improvement in the peak flow rate. We recommend conducting large scale study on the effect of yoga on asthma.
